# Boys are more stunted than girls in Sub-Saharan Africa: a meta-analysis of 16 demographic and health surveys

**DOI:** 10.1186/1471-2431-7-17

**Published:** 2007-04-10

**Authors:** Henry Wamani, Anne Nordrehaug Åstrøm, Stefan Peterson, James K Tumwine, Thorkild Tylleskär

**Affiliations:** 1 Centre for International Health, University of Bergen, Armauer Hansen Building, N-5021 Bergen, Norway; 2 Institute of Public Health, Makerere University, P.O Box 29140, Kampala, Uganda; 3 Division of International Health (IHCAR), Karolinska Institute, Norrbacka, S-17176 Stockholm, Sweden; 4 Department of Paediatrics and Child Health, Makerere University Medical School P.O Box 7072, Kampala, Uganda

## Abstract

**Background:**

Many studies in sub-Saharan Africa have occasionally reported a higher prevalence of stunting in male children compared to female children. This study examined whether there are systematic sex differences in stunting rates in children under-five years of age, and how the sex differences in stunting rates vary with household socio-economic status.

**Methods:**

Data from the most recent 16 demographic and health surveys (DHS) in 10 sub-Saharan countries were analysed. Two separate variables for household socio-economic status (SES) were created for each country based on asset ownership and mothers' education. Quintiles of SES were constructed using principal component analysis. Sex differentials with stunting were assessed using Student's t-test, chi square test and binary logistic regressions.

**Results:**

The prevalence and the mean z-scores of stunting were consistently lower amongst females than amongst males in all studies, with differences statistically significant in 11 and 12, respectively, out of the 16 studies. The pooled estimates for mean z-scores were -1.59 for boys and -1.46 for girls with the difference statistically significant (p < 0.001). The stunting prevalence was also higher in boys (40%) than in girls (36%) in pooled data analysis; crude odds ratio 1.16 (95% CI 1.12–1.20); child age and individual survey adjusted odds ratio 1.18 (95% CI 1.14–1.22). Male children in households of the poorest 40% were more likely to be stunted compared to females in the same group, but the pattern was not consistent in all studies, and evaluation of the SES/sex interaction term in relation to stunting was not significant for the surveys.

**Conclusion:**

In sub-Saharan Africa, male children under five years of age are more likely to become stunted than females, which might suggest that boys are more vulnerable to health inequalities than their female counterparts in the same age groups. In several of the surveys, sex differences in stunting were more pronounced in the lowest SES groups.

## Background

Linear growth retardation or low height-for-age, commonly known as stunting is a useful anthropometric measure for children in terms of its positive correlation with social and economic deprivation. Stunting is now acknowledged as the best proxy measure for child health inequalities [1,2]. This is because stunting captures the multiple dimensions of children's health, development and the environment where they live. Stunting is attributable to a wide range of factors [3] including low birth weight [4], inadequate care and stimulation [5], insufficient nutrition and recurrent infections [6], and other environmental determinants.

Stunting is conveniently used because empirical evidence suggests that the distribution of healthy children's height is not affected by ethnicity and race for the first five years of life [7]. Any variation between populations or ethnic groups below five years of age is due to varying degree of the growth faltering caused by factors other than genetic predisposition. The only exception is the sex difference. Thus, among well-to-do children there is a normal pattern of dimorphism where males will tend to be taller and heavier than females.

Besides studies in Asia which show higher female vulnerability [8], several studies in low-income countries have indicated that male children are more likely to be stunted than their female counterparts, most of them in sub-Saharan Africa [4,9-12]. One of our recent studies disaggregated stunting prevalence rates by sex and socio-economic status (SES) [9]. In that study it was revealed that in poorer households more boys were stunted than girls, and that the sex differences in stunting rates did not exist among children belonging to socio-economically better off groups.

In the current study we hypothesised that in many low-income countries in sub-Saharan Africa where the standard of living is generally considered low, male children will be more stunted than their female counterparts. We used data sets of 16 demographic health surveys derived from 10 sub-Saharan African countries. The aim was first, to investigate whether there exists systematic sex differences in the overall prevalence of stunting among children less than 5 years of age and second, to evaluate whether sex differences in stunting vary with household socio-economic status.

## Methods

The Demographic and Health Survey programme provides data on child anthropometric status and household-level information on mothers' education and ownership of assets for about 60 low- and middle-income countries. Population sampling frames are used for data collection, which makes the data sets nationally representative. In most countries, between 3,000 and 10,000 children below the age of 60 months are assessed for their growth status using anthropometric measurements. These data sets are in the public domain and are available from the MACRO International web-site [13]. 

We obtained data sets across sub-Saharan Africa fulfilling the following criteria: containing information on height-for-age measurements; English-speaking country, for ease of review of DHS reports; country with experience of more than one DHS study; recent surveys (conducted between 1995–2003); and data available as of September 2004. A total of 16 studies were obtained from 10 countries including Cameroon, Ghana, Kenya, Malawi, Namibia, Nigeria, Tanzania, Uganda, Zambia and Zimbabwe.

### Creation of socio-economic indices

When constructing indices for socio-economic status, one of the basic decisions concerns the domains of variables to use and methods of score. In the DHS data sets, data are available on the following domains of household wealth: characteristics of the dwelling (floor, walls, and roof material), availability of electricity, water and sanitation services, ownership of household durable goods, and parental education. Other domains that one might expect such as income or occupation are not contained in the DHS data sets.

Two variables of household SES were created reflecting the education level (mothers' education); and ownership of durable household assets and characteristics of the dwelling structure (asset index). An asset index is a good proxy for household income or expenditure [14]. An index for mothers' education was deliberately created separate from other domains because it has a known association with child health inequalities that is independent of other socio-economic indicators [9]; compared to household assets it was also considered more feasible for intervention. In addition, use of two such variables supports evidence that colinearity of many SES indicators is often too low to render them as adequate proxies for one another [9,15].

The indices were constructed separately for each country. The DHS variable on mothers' education was re-categorised by maintaining categories of "no education" (zero years of schooling) and "primary education" (not more than 7 or 8 years of schooling, depending on country). However, we merged the categories of "higher education" and "secondary education" into one category of "secondary education" (more than 7 or 8 years of schooling) because of the low numbers in the former category that would not allow for any realistic analyses. A new variable with 3 categories was thus created, (0) no education, (1) primary education and (2) secondary education.

The asset index was developed by use of principal components analysis [14] with variables on asset ownership (bicycle, radio, television, motorcycle, car/truck) and materials of the dwelling structure (floor, wall, roof) as appropriate. Regression factor scores generated from the first principal component were ranked in ascending order and then categorised into quintiles (1) poorest, to (5) least poor, Table 1, similarly presented elsewhere [16]. However, the covariance among asset variables for Tanzania 1996 and Zambia 2001/02 studies was too high to permit auto-categorisation into quintiles, instead quartiles were generated.

### Data Analysis

Statistical analyses were performed with SPSS 12.0 and STATA 8.0. Stunting was defined as height-for-age Z-score less than -2 standard deviations of the WHO/NCHS reference standards [17]. Anthropometric data were missing on approximately 25% of children. This is because children whose months and year of birth are not known for certain reasons or parents who refuse to have their children measured are excluded from anthropometric analyses in the DHS data sets. Children with incomplete data on stunting plus the flagged cases were therefore excluded from our analyses. Student's t-test, Fisher's exact test or *χ*^2 ^test, as appropriate, and logistic regressions were used to compare the outcome between male and female children. The test of homogeneity between studies was conducted and the Cochran's statistic was also reported. Results of fixed effects models are presented. Interaction between SES and gender with respect to the stunting outcome was assessed by simultaneously controlling for the main effects and the product of SES and gender in logistic regression. The level of statistical significance for all analyses was set at p < 0.05 with two-tailed comparisons.

## Results

**Figure 1:**
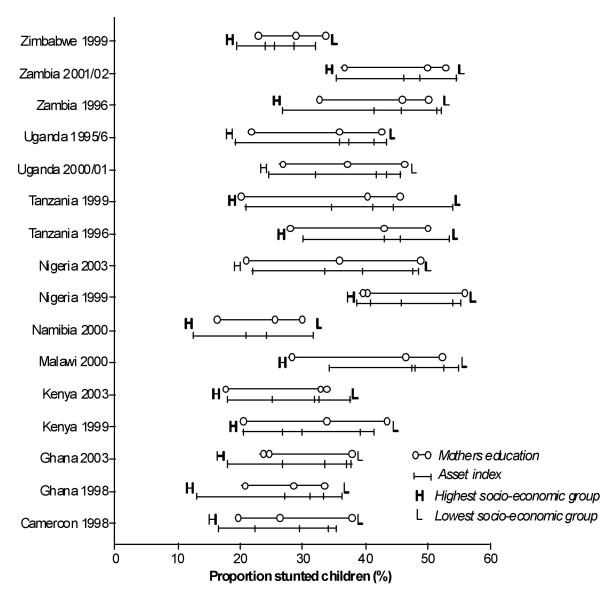
Prevalence of stunting in each of the asset index groupings (vertical bars) and in each of three groups of mothers' education (open circles) in the 16 studies.

The two SES indicators that were used – asset index and mothers' education – were almost similar in demonstrating the span and magnitude of stunting in socio-economic groupings. Generally they both showed a dose-response relationship of stunting with SES (Figure 1). However for many studies, this relationship featured a skewed pattern with poorer categories disproportionately more affected. Among the better off or the "least poor" the lowest prevalence of stunting was observed for Ghana 1998 (12%) and Namibia 2000 (13%), but was otherwise below 30% in all studies except for Nigeria 2000 (38%), Malawi 2000 (34%) and Tanzania 1996 (30%). Among quintiles and quartiles for the "most poor" the lowest prevalence of stunting was observed for Namibia 2000 (31%); and was above 50% in six studies (Malawi 2000, Nigeria 1999, Tanzania 1996 and 1999, and Zambia 1996 and 2001/02). Among mothers with no formal education the prevalence rate of stunting averaged 44% while it was 24% in those with secondary education.

The proportion of male and female children included in the analysis was nearly equal (Table 2). The mean z-scores for males were consistently lower than for females with the differences statistically significant in 12 out of 16 studies. The pooled mean z-scores (standard deviation) were -1.59 (1.56) for boys and -1.46 (1.57) for girls, and the mean difference was significant (p < 0.001).

**Figure 2:**
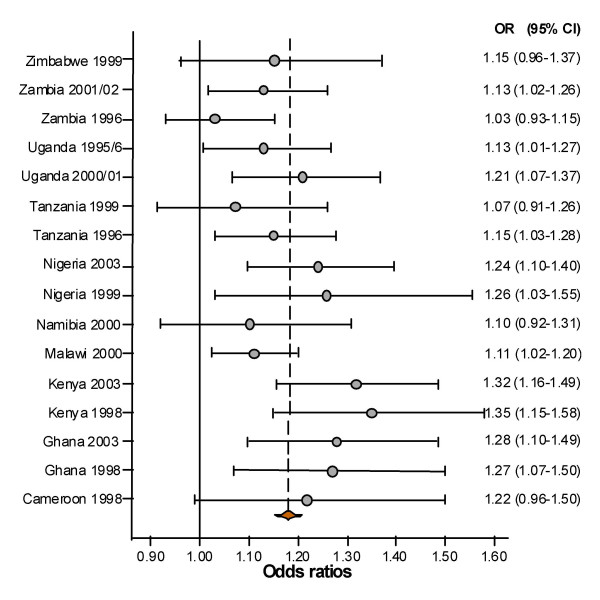
Forest plot of 16 studies indicating the excess of stunting prevalence in male compared to female children. The broken vertical line represents the odds ratio of the pooled results adjusted for child-age and individual study, with the confidence interval corresponding to the width of the diamond. The unbroken vertical line is at the null value (1) of the odds ratios (equivalent to no stunting difference between boys and girls).

The average prevalence of stunting was also higher in male than in female children in all the studies. The corresponding odds ratios (OR) for the prevalence of stunting among males compared to females were statistically significant in 11 of the 16 studies (Figure 2). In the pooled analysis the prevalence of stunting amongst males (40%) remained significantly greater than for females 36% (p < 0.001), OR 1.16 and 95% confidence interval (CI) 1.12 – 1.20. When the age of child and individual study/country were controlled for in the analysis, the adjusted OR was 1.18, CI 1.14 -1.22; and the test of homogeneity for the different studies was not significant (p = 0.15), Cochran's statistic *χ*^2 ^of 85.5 (p < 0.001); implying that studies were similar or random and fixed effects models are indistinguishable.

**Figure 3:**
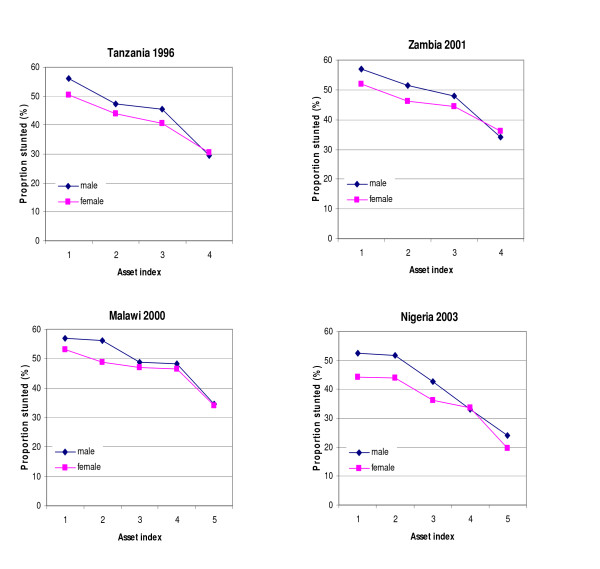
Four examples of studies with male stunting being more pronounced in the poorest socio-economic group whereas among the least poor stunting is more or less equal in the 2 sexes.

The magnitude of stunting prevalence in both sexes varied systematically and inversely with SES. The gradient depicted highly significant p-values for tests of trend in both sexes (Table 3). There was a unique pattern that was observed, although not entirely consistent across countries, for sex differences in stunting to be more pronounced among children in the poorest 2 asset quintiles and in children of mothers with no education or primary education. Figure 3, depicts graphically four examples of studies with the sex difference being more pronounced in the poorest quintiles or quartiles whereas there is no difference in socio-economically better off groups. However, sex differentials of stunting with SES did not follow a similar pattern in studies of the same country. For example the trend for Zambia 2001/2 in Figure 3, does not apply to Zambia 1996, and the trend for Tanzania 1996 does not apply to Tanzania 1999. Additionally, evaluation of the interaction term between sex and SES in relation to stunting was not statistically significant for the individual study and for the pooled analysis.

## Discussion

This systematic analysis of nationally representative datasets included 16 studies from 10 countries with a total of 64,000 children. This is the first systematic analysis of sex differences in stunting among children less than 5 years of age. Our findings demonstrate that across the 10 countries in sub-Saharan Africa, male children are consistently more likely to become stunted than their female counterparts. Secondly, in several of the studies, sex differences in stunting were more pronounced, albeit inconsistent, in the lower socio-economic strata.

In meta-analysis, two main assumptions could be employed in interpreting the effect size or the systematic difference in stunting between sexes. First, studies were drawn from a common population, and therefore share a common effect size (fixed effects model). Second, studies were drawn from populations that differ from each other in ways that could impact on effect sizes (random effects model). In the former scenario, effect size varies from one study to the next due to random error inherent in each study. In the latter scenario it varies due to both random error and true variation in effect size from one study to the next. If studies are homogeneous (significant p-value implies no homogeneity), it implies that fixed effects and random effects models are similar and not statistically different. This further implies that similar studies done in similar set-ups would yield statistically similar results with any study-to-study dispersion attributable to random error.

Turning to our findings the studies were homogeneous (p = 0.15), thus similar studies done in similar set-ups would likely yield similar results. So the next question is which countries are similar to these 10 Anglophone countries? Our understanding is that these countries share a lot of communalities with the rest of the countries in sub-Saharan Africa.

So then, was it an accidental observation or a reality that the pattern of stunting differentials between sexes across socio-economic strata was not consistent in all surveys? We believe this is a reality. First, it seems that the effect of SES on the pattern of stunting between sexes is no longer present in better off populations like Namibia. Second, it could be attributable to potential biases. On average 25% of data on stunting was missing in all the studies. Therefore, the tendency for more boys than girls being stunted in the 2^nd ^quintile rather than in the 1^st ^quintile (poorest 20%) as observed for many studies in Table 3, could be attributable to bias due to non-random loss of participants – implying that among the flagged and missing data cases, the proportion of stunted boys might have been higher than that for girls. Another possibility is misclassification of SES in some surveys leading to a dilution of the associations.

Theoretically, there could be other sources of bias in the study. First, systematic errors with the measure could lead to the observed systematic sex differences. The NCHS/WHO growth reference [17] has separate references for males and females, thus observed sex difference might be related in some way to the reference itself. If this were true however, it would be difficult to understand why the inequality differentials of stunting with sex disappear in the socio-economically better off groups in many of the studies. In future it would be interesting to repeat such a study using the newly developed WHO international growth standard [18].

Second, analysis of this study was based on a single age group (0–5 years) although growth and nutritional profiles are drastically changing over this period of time. For instance, wasting is often peaking early in the second year of life, while stunting is often increasing over the whole age period. In a separate analysis we checked for differences in sex ratio in five different age categories (0–5 years), but no significant difference was found (data not shown). Thus, the potential bias of having more boys in older ages which could artificially increase the prevalence of stunting in boys in the group was dispelled.

Third, several statistical tests were employed in the current study thereby inflating the likelihood of finding spurious associations. This limitation should be borne in mind especially when making inferences based upon the reported p-values.

Although there is paucity of studies which have systematically addressed differentials of sex with respect to health inequality in the early childhood period, sex differences in anthropometry with females having an advantage over males have been previously reported [9-12,19,20]. Speculation on the observed sex differences in these studies mainly centres on behavioural patterns. For instance in an extensive analysis of gender bias in undernutrition in sub-Saharan Africa, Svedberg proposed that the slight anthropometric advantage shown by girls, women, or both in many countries may suggest a historical pattern of preferential treatment of females due to the high value placed on women's agricultural labour [19]. On the basis of a study of gender biases among the Mukogodo of Kenya, Cronk [20] suggested that favouritism towards daughters occurred as a result of lowered socio-economic status. However, there are also studies that report greater social valorisation of sons at the detriment of daughters [21], including dietary discrimination [22], thereby dispelling conclusions of a nutritionally advantaged position of female over male children.

An alternative hypothesis of the cause of the difference is a biological explanation. Epidemiological studies in neonatology and in cohorts of pre-term infants and children, depict both morbidity and mortality to be consistently higher in males than females in early life, with the differences persisting after adjusting for gestational age and body size, and being more marked in the pre-term subjects [23-26]. Aside from the specific sex-chromosome factors, the underlying mechanisms to why male gender is associated with increased neonatal mortality and morbidity is poorly understood [26,27]. However, the reported male predominance in both symptomatic and asymptomatic morbidity [27] suggest that boys generally, are more vulnerable, which could partly explain our findings.

In evolutionary theory, selective male mortality has been previously linked to biased sex ratios at birth as modelled by Trivers and Willard [28]. The theory has been expanded by Wells [29] to explore its significance for differential sex morbidity and mortality among the under-fives. According to the expanded theory, in its summarised and simple form, natural selection favours a sex ratio of 1.0. Since there is an excess of males at conception the theory predicts mortality and morbidity to remain greater in males than in females for any given degree of environmental stress in the first 4 years of life. This theory in some way predicts our findings.

## Conclusion

This study reveals that in 10 countries in sub-Saharan Africa, male children below five years of age are more likely to become stunted than their female counterparts. An inconsistent pattern was observed where sex differences in stunting tended to be more pronounced in the poorest, socio-economically. The study also indirectly reaffirms that stunting, a proxy for child health inequalities, is as well a proxy for socio-economic inequalities. Even though the study advances knowledge on the understanding of early childhood health inequalities, it raises interesting issues that mandate further research.

### The research agenda

The sex difference in stunting with regard to SES seems not to be uniform across populations and the determinants for its variation are unknown at this stage. Further research is therefore needed to confirm and/or obtain explanation regarding sex differentials with stunting across socio-economic strata. As demonstrated in one of our previous papers [9], SES is not unidimensional. At present we do not know the number of dimensions that are critical for stunting in different contexts. In order to have effective interventions along the socio-economic pathway, researchers are urged to use a number of socio-economic indicators such as parents education, income, household dependency ratio, land and asset ownership independently, rather bunching them together as each could have its unique contribution [15,30]. Studies therefore need to decompose SES in order to identify which components are most associated with stunting differentials of sex in the different contexts.

In addition, the study findings need to be corroborated with findings from other regions. For instance many Asian populations actually suffer severe stunting in young age. Unfortunately many DHS data sets from Asia lack information on height-for-age, for example Indonesia 1997 and Philippines 1998. Even where information on height-for-age exists in the datasets such as Pakistan and Sri Lanka, missing information goes as high as 50%. It was therefore not possible to make any comparisons.

This first systematic analysis of sex differences in stunting might also serve as an eye opener for an attempt to map out vulnerability of different sexes across the lifecycle. Functional and long-term consequences associated with early male vulnerability need to be explored. This is especially important in studies for early nutrition conditions and later risk of disease, especially cardio-vascular diseases and diabetes [31], life expectancy and certain behaviours that are particularly known to be more prevalent amongst males than females. Finally, a question that mandates further research following findings of this study is the biological explanation as to why male children should be worse off compared to female children!

## Competing interests

The author(s) declare that they have no competing interests.

## Authors' contributions

HW performed the statistical analysis and drafted the manuscript. All authors participated in the design of the study, read and approved the final manuscript.

## Pre-publication history

The pre-publication history for this paper can be accessed here:



## Figures and Tables

**Table 1:** Asset quintiles and quartiles generated from scores of the first principal component

Socio-economic status	Zimbabwe 1999	Zambia 2001/2	Zambia 1996	Uganda 2000/1	Uganda 1995/6	Tanzania 1999	Tanzania 1996	Nigeria 2003
1^st^/poorest	624 (24%)	1826 (34%)	624 (11%)	1026 (20%)	993 (22%)	455 (20%)	1769 (34%)	918 (22%)
2^nd^	607 (23%)		1690 (31%)	974 (19%)	883 (20%)	469 (20%)		688 (16%)
3^rd^	286 (11%)	1547 (29%)	687 (13%)	1248 (25%)	709 (16%)	460 (20%)	1421 (28%)	1003 (23%)
4^th^	655 (25%)	936 (17%)	1503 (28%)	915 (18%)	1026 (23%)	474 (20%)	850 (17%)	710 (17%)
5^th^/wealthy	457 (17%)	1092 (20%)	949 (17%)	938 (18%)	861 (19%)	474 (20%)	1078 (21%)	917 (22%)
Total	2629	5401	5453	5101	4772	2332	5118	4236

	Nigeria 1999	Namibia 2000	Malawi 2000	Kenya 2003	Kenya 1998	Ghana 2003	Ghana 1998	Cameroon 1998

1^st^/poorest	201 (15%)	586 (20%)	2187 (24%)	920 (20%)	573 (20%)	457 (15%)	528 (20%)	324 (20%)
2^nd^	283 (20%)	575 (20%)	1095 (12%)	1258 (27%)	553 (19%)	852 (28%)	798 (30%)	361 (21%)
3^rd^	244 (17%)	473 (16%)	1605 (18%)	899 (19%)	644 (23%)	590 (19%)	190 (08%)	321 (19%)
4^th^	316 (23%)	678 (24%)	2307 (25%)	764 (16%)	598 (21%)	590 (19%)	621 (24%)	328 (20%)
5^th^/wealthy	356 (25%)	577 (20%)	1932 (21%)	848 (18%)	496 (17%)	597 (19%)	482 (18%)	329 (20%)
Total	1400	2889	9126	4689	2864	3086	2619	1663

**Table 2:** The 16 Demographic and Health Surveys (DHS) studied; with country, year of study, age groups and total number of children included in this analysis.

Country	Year of Study	Age in months	Total^a^		Mean Z-scores^a^		P-value
			Male	Female	Male	Female	
Zimbabwe	1999	0–59	1335	1297	-1.16 (1.5)	-0.97 (1.6)	0.002
Zambia	2001/02	0–59	2723	2707	-1.94 (1.5)	-1.84 (1.6)	0.011
Zambia	1996	0–59	2688	2815	-1.80 (1.5)	-1.74 (1.5)	0.147
Uganda	2000/01	0–59	2548	2597	-1.62 (1.5)	-1.55 (1.4)	0.058
Uganda	1995/6	0–47	2204	2315	-1.57 (1.5)	-1.38 (1.5)	< 0.001
Tanzania	1999	0–59	1272	1242	-1.71 (1.3)	-1.64 (1.3)	0.170
Tanzania	1996	0–59	2668	2558	-1.83 (1.4)	-1.70 (1.5)	0.002
Nigeria	2003	0–59	2165	2128	-1.55 (1.8)	-1.39 (1.8)	0.002
Nigeria	1999	0–35	739	717	-1.76 (2.1)	-1.50 (2.1)	0.017
Namibia	2000	0–59	1472	1437	-1.03 (1.4)	-0.91 (1.4)	0.019
Malawi	2000	0–59	4557	4605	-1.88 (1.6)	-1.75 (1.6)	< 0.001
Kenya	2003	0–59	2366	2353	-1.29 (1.5)	-1.08 (1.6)	< 0.001
Kenya	1998	0–35	1451	1448	-1.29 (1.7)	-1.15 (1.6)	0.026
Ghana	2003	0–59	1567	1527	-1.42 (1.4)	-1.20 (1.5)	< 0.001
Ghana	1998	0–59	1288	1338	-1.26 (1.5)	-1.12 (1.5)	0.026
Cameroon	1998	0–59	905	879	-1.17 (1.6)	-1.03 (1.5)	0.056
***All studies***	***1995 to 2003***	***0–59***	***31948***	***31963***	***-1.59 (1.6)***	***-1.46 (1.6)***	***< 0.001***

^a^Values include only children with complete data on height and age, and who were not flagged

In addition a comparison of the mean z scores (standard deviation) for height-for-age between male and female children, and p-values for the difference is indicated.

**Table 3:** A comparison of the proportion of stunted children among males and females in each of the asset index quintile from 1^st^(poorest) to 5^th^(least poor) and in each of the mothers' education groups.

Country (year of study)	Sex	Percentage stunted by household asset index quintile					p-value for test of trend	Percentage stunted by mothers education status			p-value for test of trend
					
		1^st^	2^nd^	3^rd^	4^th^	5^th^		Nil	Primary	Secondary	
Zimbabwe (1999)	M	33.1	28.4	27.3	24.2	24.3	*0.007*	31.0	31.9	22.7	*0.001*
	F	30.6	28.9	23.7	23.8	14.5	*0.001*	36.9	25.9	21.7	*0.001*
*Pearson χ*^2^*test*		*0.49*	*0.90*	*0.46*	*0.91*	*0.01*		*0.35*	*0.02*	*0.67*	
Zambia (2001/02)^b^	M	57.0		51.5	47.9	34.1	*< 0.001*	53.8	52.4	36.1	*< 0.001*
	F	52.0		46.2	44.5	36.1	*< 0.001*	52.2	47.5	35.9	*< 0.001*
*Pearson χ*^2^*test*		*0.03*		*0.04*	*0.30*	*0.48*		*0.64*	*< 0.01*	*0.95*	
Zambia (1996)	M	49.0	51.9	47.0	43.5	28.3	*< 0.001*	50.0	46.5	33.4	*< 0.001*
	F	54.1	52.4	44.3	39.3	25.2	*< 0.001*	51.1	45.4	30.9	*< 0.001*
*Pearson χ*^2^*test*		*0.20*	*0.83*	*0.48*	*0.11*	*0.28*		*0.76*	*0.53*	*0.37*	
Uganda (2000/01)	M	46.9	45.9	44.0	30.8	26.7	*< 0.001*	46.7	39.4	26.7	*< 0.001*
	F	44.2	41.1	39.5	33.1	22.5	*< 0.001*	46.7	34.9	25.7	*< 0.001*
*Pearson χ*^2^*test*		*0.38*	*0.13*	*0.11*	*0.45*	*0.14*		*0.99*	*< 0.01*	*0.77*	
Uganda (1995/6)	M	44.7	44.9	42.2	35.9	21.6	*< 0.001*	44.6	38.9	23.2	*< 0.001*
	F	42.1	38.1	32.4	35.8	16.9	*< 0.001*	41.6	32.9	19.3	*< 0.001*
*Pearson χ*^2^*test*		*0.40*	*0.04*	*< 0.01*	*1.00*	*0.08*		*0.30*	*< 0.01*	*0.21*	
Tanzania (1999)	M	54.7	47.3	37.8	36.9	22.6	*< 0.001*	44.9	42.4	19.7	*< 0.001*
	F	53.1	41.9	44.9	32.6	19.0	*< 0.001*	47.0	38.1	19.9	*< 0.001*
*Pearson χ*^2^*test*		*0.67*	*0.31*	*0.23*	*0.28*	*0.35*		*0.59*	*0.09*	*0.98*	
Tanzania (1996)^b^	M	56.2		47.2	45.5	29.6	*< 0.001*	52.9	44.3	31.0	*< 0.001*
	F	50.5		44.0	40.7	30.5	*< 0.001*	48.1	41.7	24.2	*< 0.001*
*Pearson χ*^2^*test*		*0.01*		*0.22*	*0.16*	*0.74*		*0.06*	*0.12*	*0.22*	
Nigeria (2003)	M	52.4	51.8	42.8	33.1	24.1	*< 0.001*	52.7	39.6	21.6	*< 0.001*
	F	44.2	44.0	36.2	33.7	19.6	*< 0.001*	45.9	32.1	19.6	*< 0.001*
*Pearson χ*^2^*test*		*0.01*	*0.04*	*0.03*	*0.85*	*0.11*		*< 0.01*	*0.01*	*0.38*	
Nigeria (1999)	M	58.2	54.4	47.2	46.8	41.1	*0.002*	57.4	43.2	43.4	*0.002*
	F	52.4	53.6	44.4	34.8	36.2	*0.001*	55.2	36.6	34.6	*0.001*
*Pearson χ*^2^*test*		*0.41*	*0.88*	*0.66*	*0.03*	*0.34*		*0.68*	*0.17*	*0.04*	
Namibia (2000)	M	30.7	23.0	23.8	20.7	13.9	*< 0.001*	29.8	26.7	17.0	*< 0.001*
	F	31.4	24.3	17.2	20.3	10.3	*< 0.001*	30.7	24.3	14.4	*< 0.001*
*Pearson χ*^2^*test*		*0.87*	*0.72*	*0.08*	*0.89*	*0.19*		*0.84*	*0.38*	*0.20*	
Malawi (2000)	M	57.0	56.2	48.8	48.2	34.6	*< 0.001*	53.7	48.3	28.7	*< 0.001*
	F	53.2	48.9	47.1	46.4	34.0	*< 0.001*	52.2	45.1	26.7	*< 0.001*
*Pearson χ*^2^*test*		*0.07*	*0.01*	*0.48*	*0.40*	*0.78*		*0.44*	*< 0.01*	*0.57*	
Kenya (2003)	M	39.6	35.7	34.4	25.8	24.0	*< 0.001*	36.2	35.7	20.8	*< 0.005*
	F	35.2	29.3	29.1	24.1	11.5	*< 0.001*	31.8	29.8	13.7	*< 0.001*
*Pearson χ*^2^*test*		*0.18*	*0.02*	*0.08*	*0.58*	*0.01*		*0.17*	*< 0.01*	*< 0.01*	
Kenya (1998)	M	46.0	42.3	28.2	31.7	25.5	*< 0.001*	44.5	37.2	24.0	*< 0.001*
	F	37.0	35.9	25.3	27.9	15.3	*< 0.001*	42.9	30.9	16.0	*< 0.001*
*Pearson χ*^2^*test*		*0.03*	*0.10*	*0.41*	*0.31*	*0.01*		*0.76*	*< 0.01*	*< 0.01*	
Ghana (2003)	M	39.2	40.6	29.7	36.0	20.7	*< 0.001*	41.0	29.1	25.1	*< 0.001*
	F	36.3	33.3	24.1	31.0	15.4	*< 0.001*	36.3	20.8	21.8	*< 0.001*
*Pearson χ*^2^*test*		0.52	0.03	0.13	0.20	0.11		0.06	0.01	0.12	
Ghana (1998)	M	37.8	35.1	39.4	28.9	13.8	*< 0.001*	35.8	32.8	22.2	*< 0.001*
	F	34.4	27.0	27.5	25.2	12.2	*< 0.001*	31.9	24.5	18.2	*< 0.001*
*Pearson χ*^2^*test*		*0.42*	*0.01*	*0.08*	*0.31*	*0.60*		*0.15*	*0.04*	*0.12*	
Cameroon (1998)	M	38.2	35.5	36.5	25.6	14.9	*< 0.001*	41.3	28.3	20.7	*< 0.001*
	F	32.0	32.6	22.5	18.9	18.2	*< 0.001*	35.0	24.4	17.6	*< 0.001*
*Pearson χ*^2^*test*		*0.24*	*0.56*	*0.01*	*0.14*	*0.42*		0.13	0.23	0.37	

M = Male; F = Female; ^b^The asset index in this survey was categorised in quartiles

Pearson chi square test for each male-female comparison and p-values for the test of trend across quintiles or mothers education groups is indicated.
